# Does type 2 diabetes mellitus promote intervertebral disc degeneration?

**DOI:** 10.1007/s00586-016-4612-3

**Published:** 2016-06-06

**Authors:** Stella Maris Fabiane, Kirsten J. Ward, James C. Iatridis, Frances M. K. Williams

**Affiliations:** 1 Department of Twin Research, King’s College London, St Thomas’ Hospital Campus, 4th Floor South Wing Block D, Westminster Bridge Road, London SE1 7EH, UK; 2 Leni and Peter W. May Department of Orthopaedics, Icahn School of Medicine at Mount Sinai, 1 Gustave Levy Place, Box 1188, New York, NY 10029-6574, USA

**Keywords:** Lumbar disc disease, Type 2 diabetes, Lumbar intervertebral disc degeneration

## Abstract

**Purpose:**

LDD is an important cause of low back pain. Many people believe there is an adverse influence of type 2 diabetes (T2D) on lumbar intervertebral disc degeneration (LDD). We examined a population sample for epidemiological evidence of association.

**Methods:**

Twin volunteers from the TwinsUK cohort having spine magnetic resonance (MR) scans coded for LDD and information about T2D were investigated in two ways. First, as a population sample and second as a cotwin case control study in twin pairs discordant for T2D. Other risk factors for LDD considered were age, body-mass index (BMI), smoking, and alcohol.

**Results:**

In 956 twin volunteers T2D had a prevalence of 6.6 %. LDD score was higher in T2D twins (14.9 vs 13.1 *p* = 0.04) but was not an independent risk factor if the influence of age and BMI were included in the model. Discordant twin analysis (*n* = 33 pairs) showed no significant difference in LDD between twins having T2D and their unaffected cotwins.

**Conclusions:**

Twins having T2D did manifest higher LDD scores but the effect was abrogated once BMI was included in multivariable analysis, showing it is not an independent risk factor for LDD. The population study had 80 % power at 0.1 significance level to detect a difference of 1.8 in LDD score (range of 0–60), so if there is an effect of T2D on LDD, it is likely to be small.

## Introduction

Low back pain is highly prevalent in the Western world and accounts for considerable work absenteeism. There is an accepted relationship between back pain and lumbar disc degeneration (LDD), although the strength of the association remains debated [1, 2]. There is some evidence that type 2 diabetes mellitus (T2D) is important in the aetiology of LDD. T2D is reported to be associated with spinal stenosis [3], Individuals with obesity and T2D are at an increased risk of low back pain and musculoskeletal complications but the relative contributions of the two risk factors remains unclear [4, 5].

Worldwide prevalence of diabetes mellitus in general is 9 % of the population with T2D accounting for 90 % of all the cases. Increased body-mass index (BMI) is one of the most important risk factors for T2D and an epidemic of obesity is leading to increased T2D prevalence. This has important implications for low back pain and disability, which already represent considerable social challenges. Changes in intervertebral disc physiology and structure in diabetes are well documented in animal models [6, 7] and in vitro studies of disc cells in high glucose media support a deleterious effect. Increased BMI is also a well-recognised risk factor for LDD [5, 8] although the strength of this association has been disputed [9].

A population based study of LDD epidemiology accounting for T2D has not yet been described, perhaps because of the considerable heritability of LDD (>70 % of the phenotypic variance in LDD is genetic). This means that there is considerable genetic influence on LDD phenotype variation, so large population samples are required for adequate power. The inherent genetic matching of twin pairs provides a powerful study design and in TwinsUK an unselected sample of twins have had LDD determined using the gold standard method of T2-weighted MR scans.

## Methods

TwinsUK is a large registry of same-sex twins containing both monozygotic (MZ) and dizygotic (DZ) same sex twin pairs. It contains extensive genotype and phenotype data obtained at clinical visits and by questionnaire. TwinsUK has contributed to the understanding of a wide variety of traits and diseases including musculoskeletal disease and LDD and they are similar to the general singleton population [2, 10, 11]. We examined the association between LDD and T2D status of twins having baseline lumbar spine MRI scans [10] as a population sample. In addition we considered the twin pairs in a T2D discordant co-twin design using the inherent matching within twin pairs for age, sex, genetic factors [100 % in monozygotic (MZ) and on average 50 % in dizygotic (DZ) twin pairs] and other measured and unmeasured confounders. MR scans had been scored for LDD and the summary measure of disc degeneration (LDD score) was made considering four features (disc height, disc signal intensity, disc bulge and anterior osteophytes) each coded 0–3 and summed over five discs [10]. T2D was defined by the serum fasting glucose level (≥7 mmol l^−1^) and/or self-report of a physician’s diagnosis of T2D on questionnaire, as previously [12]. Other risk factors for LDD considered were age, body mass index, smoking, alcohol consumption and glycated haemoglobin (HbA1C, mmol^−1^). Ethics permission had been obtained from the St Thomas’ Hospital ethics committee and twins gave fully informed written consent.

Statistical comparisons were made using STATA software (StataCorp, College Station, TX, USA). Univariable linear regression was adjusted for family relatedness; multivariable linear regression was adjusted for family relatedness, age, sex, BMI, smoking and alcohol consumption. Summary LDD score was compared between T2D and controls in the whole sample (using *t* test) as well as in the subset of twin pairs discordant for T2D (using Wilcoxon rank sum test).

## Results

The sample comprised 956 TwinsUK volunteers having both spine MR images and information on T2D. T2D prevalence was 6.6 % in this sample. The mean age was 54 years (range 19–73 years) and 917 (95.9 %) twin volunteers were female. The mean body mass index (BMI) was 24.8 kg/m^2^, details of the sample are shown in Table 1. The mean LDD score was 13.2 (SD = 7.7) (range 0–60; 4 MR features coded 0–3 and summed over 5 discs).

Comparing the T2D twins with unaffected twins revealed LDD to be significantly increased in T2D (14.9 vs 13.1, *t* test *p* = 0.04). Risk factors significantly associated at the 5 % level with LDD on univariable analysis were age (*p* < 0.001) BMI (*p* = 0.004) and T2D (regression adjusted for relatedness *p* = 0.02). When all available risk factors (excluding HbA1C) were included in a multivariable regression, only age (*p* < 0.001) and BMI (*p* = 0.02) remained statistically significant, suggesting that the effect of increased BMI, rather than T2D per se, was influencing LDD. If T2D was included in the model but not BMI, T2D was still not statistically significant (Table 2). We have also assessed association of glycated haemoglobin (HbA1C) with LDD. There were fewer measurements of this biomarker (*n* = 36) so we adjusted for age and BMI, with no evidence of association between LDD and HbA1C (univariable regression *p* = 0.15; multivariable regression adjusted for age, BMI *p* = 0.29) suggesting no effect of prolonged hyperglycaemia *per se*. The population study had 80 % power at 0.1 significance level to detect a difference of 1.8 in LDD score (range 0–60).

Twin pairs discordant for T2D were also considered in a smaller but more closely matched study (*n* = 33 twin pairs): there were 7 MZ pairs and 26 DZ pairs. When the LDD scores between T2D cases and controls (*n* = 33 pairs) were compared, no evidence of difference was observed (*p* = 0.90). The same finding was made if the analysis was stratified by zygosity (*p* = 0.20 for MZs; *p* = 0.54 for DZs, Table 3).

## Discussion

There are many plausible biological reasons why T2D might increase LDD via increased protein glycation, with advanced glycation end-product accumulation shown to accelerate LDD in animal models [6, 13]. This is the first epidemiological study of the association between LDD and T2D in humans. The phenotyping for LDD in this study was the gold standard T2 weighted MR scan. There is no international consensus on how degenerative change should be coded, and there is a move towards using individual MR scan features to improve biological relevance [2, 10, 11]. An initial, unadjusted, comparison between LDD scores in T2D and controls in this predominantly female sample did show greater LDD in those having T2D. When the other risk factors were taken into account, however, only age and BMI remained associated with LDD. An association was not detected with smoking, gender or alcohol consumption in this sample, although the first two have been found associated in TwinsUK [2] and other studies [14]. In linear regression T2D was not associated with LDD; the effect appeared to be mediated by BMI—this was shown by mutually excluding T2D and BMI from the regression models. That is to say, the apparent predisposition to LDD in T2D was entirely accounted for by BMI. Results are consistent with a prior investigation in nine pairs of identical male twins that found no effect of insulin-dependent (type 1) diabetes on disc degeneration [15]. While type 1 and type 2 diabetes have distinct aetiologies they both result in hyperglycaemia so may have similar influence on LDD. Together, these studies support the notion that T2D risk factors including increased BMI may have more important influence on LDD than hyperglycaemia *per se*. BMI remains a consistent risk factor in the absence of T2D, a finding consistent with others’ work [5].

The possibility of other unidentified risk factors (both environmental and genetic) confounding an association with LDD are well controlled for using a discordant twin analysis. There were only a few monozygotic pairs affected by T2D so we included dizygotic pairs as well (total *n* = 33 pairs). This analysis did not show evidence of difference in LDD between T2D cases and their co-twin controls.

There are several weaknesses to this study, with the main ones being the limited sample size, fairly low prevalence of T2D and the predominance of females in the sample, for historical reasons. The prevalence of T2D was 6.6 % in the TwinsUK sample but 9 % in the general population—per-haps reflecting relatively healthy registry volunteers. The limited differences in HbA1C between cases and controls is suggestive of pre-diabetes in controls, and is indicative of reasonably good glycaemic control in cases—so less power to detect a difference between the two. We have not adjusted for diabetic medication or factored in the degree of blood sugar control. Finally, for historical reasons TwinsUK has a small proportion of males, making it difficult comment on the influence of T2D in men.

This is the first study to investigate in humans the influence of T2D on LDD. Our data do not provide evidence of a direct effect of T2D on LDD despite the study having the power to detect small changes in summary degenerative change score on MR spine scans. Our results—based on a predominantly female population sample—suggest that the association seen is mediated by increased BMI which is a well-documented risk factor for both traits. Our work suggests that research efforts for managing low back pain should be directed not at the study of hyperglycaemia on intervertebral disc but towards the control of BMI, at least in women.

## Figures and Tables

**Table 1:** Comparison of twin cases and controls

	T2D cases	Controls	Total	*p* value
*N*	63 (6.6 %)	893 (93.4 %)	956	
Females	61 (6.4 %)	856 (89.5 %)	917 (95.9 %)	0.71
Age (SD) years	59.4 (7.3)	53.6 (8.3)	53.9 (8.4)	<0.001
BMI (SD) kg/m^2^	27.3 (5.2)	24.6 (4.1)	24.7 (4.3)	<0.001
Smoking				
Non	31 (3.2 %)	422 (44.1 %)	453 (47.4 %)	
Ex	21 (2.2 %)	246 (25.7 %)	267 (27.9 %)	0.49
Current	7 (0.7 %)	124 (13.0 %)	133 (13.7 %)	
Alcohol	2.4 (SD = 1.2)	2.8 (SD = 1.4)	2.8 (SD = 1.4)	0.03
Zygosity				
MZ	28 (2.9 %)	290 (30.3 %)	318 (33.3 %)	0.05
DZ	35 (3.7 %)	603 (63.1 %)	638 (66.7 %)	
LDD score	14.9 (SD = 6.5)	13.1 (SD = 7.7)	13.2 (SD = 7.7)	0.04

The LDD score is the summation of four features (disc height, disc signal intensity, disc bulge and anterior osteophytes) each coded 0–3, summed over the five lumbar discs. Alcohol consumption was by self-report, averaged over one week in a lifetime, in alcohol units

**Table 2:** Risk factors assessed for association with LDD, by linear regression

	Univariable				Multivariable (all variables included) *N* = 811			Multivariable, excluding T2D *N* = 811			Multivariable, excluding BMI *N* = 812		
				
	*n*	β	95 % CI	*p*	β	95 % CI	*p*	β	95 % CI	*p*	β	95 % CI	*p*
Age (years)	956	0.067	0.058–0.076	<0.001	0.071	0.061–0.081	<0.001	0.070	0.0602–0.0799	<0.001	0.071	0.061–0.081	<0.001
BMI (kg/m^2^)	948	0.031	0.010–0.052	<0.01	0.022	0.004–0.040	0.02	0.021	0.003–0.038	0.02	–		
Smoking	851												
Ex-smoker		0.132	−0.047 to 0.310	0.15	0.046	−0.117 to 0.209	0.58	0.045	−0.118 to 0.208	0.59	0.051	−0.112 to 0.214	0.54
Current		−0.205	−0.452 to 0.041	0.11	−0.088	−0.301 to 0.125	0.42	−0.089	−0.089 to 0.108	0.41	−0.091	−0.307 to 0.124	0.41
Alcohol	904	−0.054	−0.110 to 0.0019	0.06	0.029	−0.023 to 0.081	0.27	0.030	−0.022 to 0.082	0.26	0.025	−0.026 to 0.077	0.34
Sex	956	0.183	−0.249 to 0.615	0.41	−0.066	−0.472 to 0.340	0.75	−0.059	−0.461 to 0.342	0.77	−0.021	−0.433 to 0.391	0.92
T2D	956	0.305	0.0492–0.561	0.02	−0.128	−0.359 to 0.104	0.28	–			−0.060	−0.294 to 0.175	0.62

Univariable regression included the variable in the leftmost column in a single model (six models in total); multivariable regression included all variables in the leftmost column. BMI and T2D as possible co-dependent risk factors were tested by including both in the multivariable model, then by mutually excluding them. All regressions were adjusted for relatedness

*N* sample size; β estimated effect size; *CI* confidence interval

**Table 3:** LDD score in T2D cases and controls in discordant twin pairs, overall and by zygosity

	*N*	T2D cases	T2D controls	*p*
All discordant pairs LDD mean (SD)	33	14.6 (6.8)	14.8 (10.4)	0.90
MZ LDD mean (SD)	7	17.7 (7.7)	15.9 (9.1)	0.20
DZ LDD mean (SD)	26	13.7 (6.4)	14.5 (10.8)	0.54
